# Decoding calvarial lesions: Contemporary neurosurgical management integrating radiological and histopathological features

**DOI:** 10.1016/j.bas.2026.106655

**Published:** 2026-08-18

**Authors:** Biyan Nathanael Harapan, Antonia Clarissa Wehn, Viktoria Ruf, Thomas Liebig, Willy R. Hube, Luis Kuschel, Florian Ringel, Veit M. Stöcklein, Michael Schmutzer-Sondergeld

**Affiliations:** a Department of Neurosurgery, LMU University Hospital, LMU Medizin, Marchioninistraße 15, Munich, 81377, Germany; b Center for Neuropathology and Prion Research, Faculty of Medicine, LMU Medizin, Feodor-Lynen-Straße 23, Munich, 81377, Germany; c Department for Diagnostic and Interventional Neuroradiology, LMU University Hospital, LMU Medizin, Marchioninistraße 15, Munich, 81377, Germany

**Keywords:** Calvarium, Calvarial lesions, Tumor, Skull, Neurooncology, Osteolytic lesions

## Abstract

**Introduction:**

Calvarial lesions range from benign tumors to malignant processes and pose diagnostic and surgical challenges due to their rarity and heterogeneous imaging features.

**Research question:**

Which clinical and radiological features distinguish benign from malignant calvarial lesions, and how do they influence neurosurgical management and outcomes?

**Material and methods:**

We performed a retrospective study of patients undergoing neurosurgical treatment for calvarial lesions between 2013 and 2025. Clinical, radiological, surgical, and histopathological data were analyzed. Functional outcomes (KPS, GOS, mRS), comorbidity measures (ASA classification, BMI), and frailty (modified 5-Item Frailty Index) were assessed. Imaging evaluation included CT-based bone morphology and MRI.

**Results:**

A total of 174 patients were included; 66.1% had benign and 33.9% malignant lesions. Malignant lesions were associated with older age, higher frailty, and comorbidity burden. The most common diagnoses were meningioma and metastases. Multiple osteolytic lesions on CT were associated with malignancy and remained the strongest independent predictor (OR: 5.34; 95% CI: 2.35–12.14). Gross total resection was achieved in 82.8% of cases (83.5% in benign vs. 81.3% in malignant lesions, p = 0.8), with significant postoperative functional improvement (KPS), particularly in benign lesions (p < 0.0001). Overall, recurrence occurred in 3.4% of patients and was more frequent in malignant than benign lesions (6.8% vs. 1.7%, p = 0.2).

**Discussion and conclusion:**

Multiple osteolytic lesions represent a key radiological marker of malignancy and support preoperative risk stratification. Distinguishing benign from malignant lesions is essential, as it informs surgical strategy and the extent of resection, enabling individualized patient management.

## Highlights

• One of the largest neurosurgical cohorts of calvarial lesions reported to date.
• Multiple osteolytic lesions on CT indicate a high likelihood of malignancy.
• Malignant lesions associated with advanced age, frailty, and comorbidities.
• Surgical management improves functional outcomes.
• Recurrence predominantly occurs in malignant and infiltrative lesions.

## 1 Introduction

Lesions of the calvarium represent a heterogeneous group of entities that can arise from the cranial bones themselves or result from secondary infiltration by adjacent or systemic disease processes. Primary calvarial tumors are rare, accounting for approximately 1% of all primary bone tumors, and the available literature remains largely limited to isolated case reports and small series, underscoring the need for comprehensive analyses (Shuman et al., 2025; Kakkar et al., 2016). Their clinical presentation is highly variable, ranging from incidentally detected, asymptomatic findings to painful, palpable masses. Focal neurological deficits can also occur if adjacent functionally relevant intracranial structures are affected (Nasi-Kordhishti et al., 2021). While benign lesions such as osteomas, intraosseous meningiomas, and hemangiomas typically follow an indolent course, malignant or infiltrative pathologies – including metastases and plasmacytomas – may exhibit rapid progression and require multimodal therapy (Ozgiray et al., 2016; Harapan et al., 2025; Allen et al., 2018).

The characterization and classification of calvarial lesions remain challenging due to their marked biological and radiological heterogeneity (Akhaddar et al., 2025; El Asri et al., 2025). Previous studies have predominantly classified these lesions according to radiological characteristics or a benign–malignant framework (Gomez et al., 2018), but have largely focused on imaging or histopathological findings only without integrating surgical management and clinical outcomes. By applying a pragmatic benign–malignant framework, while simultaneously incorporating radiological, histopathological, surgical, and outcome data, we aim to provide a clinically oriented structure that may facilitate diagnostic reasoning and surgical decision-making.

Imaging plays a pivotal role in the initial evaluation of calvarial lesions. High-resolution computed tomography (CT) enables detailed assessment of bone architecture, allowing differentiation between osteolytic and osteoblastic changes, while magnetic resonance imaging (MRI) provides superior visualization of soft-tissue extension and dural or intracranial involvement (Gomez et al., 2018; Lloret et al., 2009). However, despite characteristic radiological patterns, the distinction between benign and malignant lesions often remains uncertain preoperatively, underscoring the necessity of histopathological confirmation for definitive diagnosis (Nasi-Kordhishti et al., 2021). This diagnostic ambiguity is of particular relevance for surgical decision-making, as the suspected biological behavior of a lesion directly influences the extent of resection, the need for reconstruction, and consideration of adjuvant therapies. Given their rarity, calvarial lesions are primarily discussed in isolated case reports or small series, with limited comprehensive analyses on larger patient cohorts. Moreover, comparative evaluations that explicitly contrast benign and malignant calvarial lesions across clinical, radiological, and surgical domains remain scarce.

To date, and to the best of our knowledge, only few comprehensive studies have systematically integrated radiological characteristics, histopathological findings, and neurosurgical management within a single analytical framework for calvarial lesions. As a result, the interplay between imaging features, pathological diagnosis, and surgical decision-making remains incompletely understood. In particular, a structured dichotomous assessment distinguishing benign from malignant lesions – while acknowledging the biological and radiological overlap between these entities – has not been consistently applied in previous investigations.

The present study therefore aims to investigate calvarial lesions from a multidisciplinary perspective, integrating imaging analysis, histopathological evaluation, and neurosurgical treatment strategies. By combining these complementary domains we seek to improve individualized risk stratification, optimize surgical planning, and ultimately enhance patient-specific outcomes. A central focus is placed on the comparative analysis of benign versus malignant lesions throughout all stages of evaluation and treatment, employing a pragmatic dichotomous framework that is not intended to be absolute but rather clinically informative.

## 2 Material and methods

### 2.1 Study design and objectives

This retrospective observational study aimed to analyze the clinical, radiological, and surgical characteristics of patients who underwent neurosurgical intervention for calvarial lesions at the Department of Neurosurgery, LMU University Hospital in Munich between January 2013 and May 2025. The primary objective was to characterize the clinical presentation, imaging features, histopathological spectrum, and outcomes following neurosurgical management. Secondary objectives included complication rates and the influence of patient-related and lesion-specific factors on recurrence and overall outcome.

### 2.2 Patient selection and inclusion criteria

All patients who received surgical treatment for a calvarial lesion within the defined study period were eligible for inclusion. Cases were identified through a systematic review of the institutional neurosurgical database. Inclusion criteria comprised: Histopathologically defined primary or secondary calvarial lesion, surgical intervention performed at the Department of Neurosurgery, LMU University Hospital, availability of pre- and postoperative neuroimaging and complete clinical follow-up data. Patients with insufficient imaging, missing histopathological confirmation, or incomplete follow-up documentation were excluded.

Importantly, this study specifically reflects a surgically treated cohort and therefore represents lesions that were considered to require neurosurgical evaluation and intervention. Patients managed conservatively or treated exclusively in other specialties (e.g., oncology, hematology, dermatology) were not captured in the present dataset. Consequently, the study does not include the full spectrum of calvarial lesions encountered in clinical practice, particularly those with clear non-surgical management pathways. Lesions were included irrespective of whether they originated primarily from the calvarial bone or represented secondary osseous involvement by adjacent or systemic disease. However, inclusion required that the calvarial component constituted a relevant target of neurosurgical evaluation or intervention. Purely intracranial tumors without meaningful osseous involvement were not included.

### 2.3 Data collection and clinical parameters

All clinical, radiological, and histopathological data were obtained retrospectively from institutional electronic medical records, surgical reports, and radiology archives. The following parameters were extracted for each patient:

Demographic and surgical data: age, sex, date of surgery, preoperative symptoms, lesion side (right/left), number of neurosurgical procedures, time interval to reoperation where applicable.

**Fig. 1:**
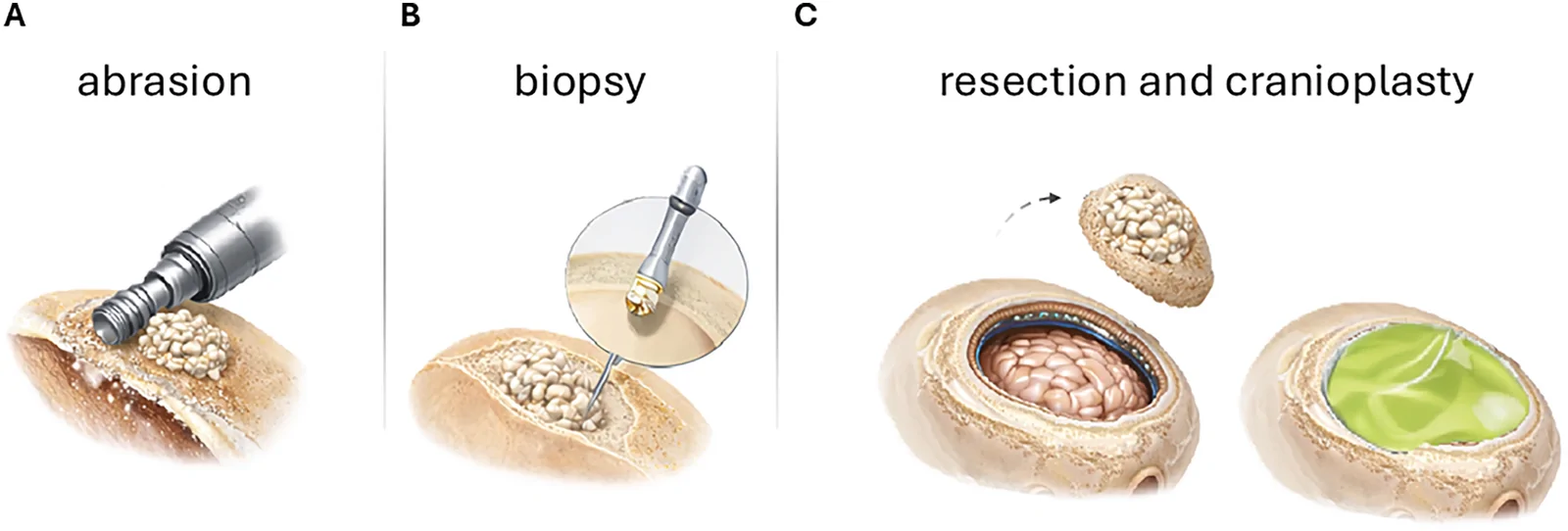
Schematic illustration of the principal surgical strategies: (A) lesion abrasion, representing limited removal or contouring of the osseous lesion while preserving the surrounding bone; (B) biopsy only, performed for diagnostic tissue sampling when definitive resection is not indicated or feasible; (C) craniotomy and lesion resection with removal of the affected calvarial segment and cranioplasty, demonstrating reconstruction of the skull defect following resection.

Surgical characteristics: dural reconstruction (yes/no), meningeal infiltration (yes/no), venous sinus infiltration (yes/no), postoperative complications (1 = hemorrhage, 2 = infection, 3 = other), and administration of adjuvant therapy (radiation and/or systemic therapy). Meningeal and venous sinus infiltration were assessed exclusively based on preoperative neuroradiological imaging (CT and/or MRI), including evidence of dural involvement and invasion of the adjacent venous sinuses. These variables were derived solely from imaging findings and were not determined by intraoperative assessment or histopathological examination. In addition, the surgical modality was systematically recorded and categorized as lesion abrasion without craniotomy, biopsy only, or craniotomy with lesion excision (Fig. 1). Biopsies were performed exclusively in selected lesions with a high preoperative suspicion of malignancy, when tissue confirmation was required for treatment planning or when primary complete resection was not considered appropriate due to lesion extent, anatomical considerations, or the need for individualized oncological management.

Follow-up data: recurrence (yes/no), date of recurrence, last follow-up date, follow-up duration (days/months), and death (yes/no). Recurrence was defined as radiological evidence of newly developed or progressive lesion following previous surgical treatment. In all cases classified as recurrence, the imaging findings prompted repeat neurosurgical intervention, and recurrence was subsequently confirmed histopathologically after re-resection.

Imaging parameters: CT morphology categorized as osteolytic, osteoblastic, combined osteolytic/osteoblastic, or no pathological osseous changes. MRI signal intensity on T1-weighted images was classified as hypointense, isointense, or hyperintense. MRI signal intensity on T2-weighted images was classified as hypointense, isointense, or hyperintense. Contrast enhancement on MRI was recorded as present or absent.

Functional and outcome measures: Karnofsky Performance Status (KPS), Glasgow Outcome Scale (GOS), and modified Rankin Scale (mRS), each assessed preoperatively and postoperatively.

Histopathological and proliferative markers: definitive histopathological diagnosis.

Comorbidity and frailty assessment: body mass index (BMI), American Society of Anesthesiologists (ASA) classification, and the modified 5-Item Frailty Index (mFI-5), incorporating the presence of arterial hypertension, congestive heart failure, chronic obstructive pulmonary disease (COPD), diabetes mellitus, and non-independent preoperative functional status. Each item within the mFI-5 was assigned one point, yielding a total score between 0 and 5. Based on the cumulative score, patients were categorized into four frailty groups: mFI-5 = 0 (not frail), mFI-5 = 1 (mild frailty), mFI-5 = 2 (moderate frailty), and mFI-5 ≥ 3 (severe frailty) (Harapan et al., 2026; Chung et al., 2024; Li et al., 2024).

### 2.4 Imaging analysis

Preoperative and postoperative imaging included cranial computed tomography (CT) and magnetic resonance imaging (MRI), performed using standard institutional protocols with 1.5- or 3.0-T scanners (Magnetom Symphony or Magnetom Skyra, Siemens Healthineers, Erlangen, Germany). CT scans were evaluated for bone morphology (osteolytic, osteoblastic, or mixed changes) and extent of osseous destruction. For osteolytic lesions, additional subclassification was performed according to lesion number (solitary vs. multiple) and the extent of bony involvement. Osteolytic lesions were classified as partial when limited to one or more components of the calvarial structure (internal table, diploë, or external table) without complete penetration. Lesions were classified as complete when the osteolysis resulted in full-thickness destruction of the calvarial bone, involving the internal table, diploë, and external table. In cases with multiple osteolytic lesions, the entity was considered to exhibit complete calvarial destruction if at least one lesion demonstrated full-thickness involvement of the calvarial bone, even when other lesions showed only partial bony destruction.

MRI protocols consisted of axial, sagittal, and coronal T1-weighted sequences before and after intravenous administration of gadolinium-based contrast agent (0.1 mmol/kg body weight), as well as T2-weighted and fluid-attenuated inversion recovery (FLAIR) sequences.

### 2.5 Histopathological analysis

All surgical specimens obtained during neurosurgical intervention underwent histopathological examination according to standard institutional protocols. Definitive diagnoses were established based on microscopic evaluation, supplemented by immunohistochemistry and, where applicable, molecular analyses, in accordance with current WHO classification criteria. Histopathological findings served as the reference standard for lesion classification and were correlated with preoperative imaging characteristics, surgical findings, and clinical outcomes.

### 2.6 Statistical analysis

Statistical analyses were performed using GraphPad Prism version 11.0 (GraphPad Software, San Diego, CA, USA). Continuous variables are reported as mean ± standard deviation and were compared using Student's t-test or the Mann–Whitney *U* test, as appropriate. Comparisons across multiple groups were performed using the Kruskal–Wallis test. Categorical variables were analyzed using the chi-square or Fisher's exact test. Associations between variables were first explored in univariate analyses, and relevant factors were subsequently included in multivariate regression models to identify independent predictors. Correlation analyses were performed using Pearson or Spearman coefficients, depending on data distribution. A two-sided p-value < 0.05 was considered statistically significant.

### 2.7 Ethical considerations

This study was conducted in accordance with the ethical standards of the Declaration of Helsinki (2013 revision). The study retrospectively analyzed patients treated at the Department of Neurosurgery, LMU University Hospital Munich, between January 2013 and May 2025. Ethical approval was obtained from the Ethics Committee of the LMU Munich (Ethikkommission der LMU München) under project number 25-1012, with approval granted on December 18, 2025, thereby covering the entire study period. All data were pseudonymized prior to analysis, and no patient-identifiable information was accessible to investigators outside the clinical care team. Data collection and storage were performed exclusively on secure hospital servers within the LMU network infrastructure. Imaging data were obtained as part of routine diagnostic and follow-up procedures and were analyzed retrospectively for research purposes.

## 3 Results

### 3.1 Patient characteristics

A total of 174 patients who underwent neurosurgical treatment for calvarial lesions were included in the final analysis. The cohort comprised 102 female patients (58.6%) and 72 male patients (41.4%), with a mean age of 48.7 ± 21.4 years (range: 2–91 years).

Based on histopathological diagnosis, 115 lesions (66.1%) were classified as benign, while 59 lesions (33.9%) were malignant. Patients with malignant lesions were significantly older than those with benign lesions (63.6 ± 16.9 vs. 41.2 ± 19.4 years, *p* < 0.0001) (Table 1). Frailty assessment using the mFI-5 revealed that 132 patients (75.9%) were not frail, 33 (19.0%) mildly frail, 7 (4.0%) moderately frail, and 2 patients (1.1%) severely frail. All variables required for calculation of the modified 5-Item Frailty Index (mFI-5) were available for all patients (no missing values). Malignant lesions were significantly associated with higher frailty scores (p < 0.0001). The mean body mass index was 24.1 ± 4.9 kg/m^2^, without significant differences between benign and malignant groups (p = 0.4). Higher ASA classifications were significantly more frequent in patients with malignant lesions (*p* < 0.0001).

**Table 1:** Baseline demographic characteristics, clinical presentation, and treatment-related parameters of patients with calvarial lesions, stratified by benign versus malignant histopathological classification.

*Parameters*	Benign lesion	Malignant lesion	*p-value*
***Patient characteristics***			
**Total, n (%)**	115 (66.1)	59 (33.9)	
**Sex, n (%)**			
Male	45	27	0.4
Female	70	32	
**Age (years), mean ± SD**	41.2 ± 19.4	63.6 ± 16.9	**<0.0001**
**KPS, mean ± SD**			
Preoperative	89.5 ± 6.9	83.2 ± 12.6	**<0.0001**
Postoperative	98.0 ± 5.3	87.6 ± 14.4	**<0.0001**
**mRS, mean ± SD**			
Preoperative	0.9 ± 0.5	1.3 ± 0.9	**0.0006**
Postoperative	0.1 ± 0.4	1.0 ± 1.2	**<0.0001**
**GOS, mean ± SD**			
Preoperative	5.0 ± 0.2	4.6 ± 0.6	**<0.0001**
Postoperative	5.0 ± 0.2	4.6 ± 0.6	**<0.0001**
**mFI-5, n (%)**			
Not frail	100 (87.0)	32 (54.2)	**<0.0001**
Mild frailty	13 (11.3)	20 (33.9)	**0.0008**
Moderate frailty	2 (1.7)	5 (8.5)	**0.04**
Severe frailty	0 (0)	2 (3.4)	0.1
**BMI (kg/m^2^), mean ± SD**	23.8 ± 5.0	24.6 ± 4.6	0.4
**ASA classification, n (%)**			
I	24 (20.9)	0 (0)	**<0.0001**
II	62 (53.9)	12 (20.3)	**<0.0001**
III	29 (25.2)	43 (72.9)	**<0.0001**
IV	0 (0)	4 (6.8)	**0.01**
V	0 (0)	0 (0)	0.99
VI	0 (0)	0 (0)	0.99
**Entity, n (%)**	Meningioma: 30 (26.1) Osteoma: 22 (19.1) Langerhans cell histiocytosis: 24 (20.9) Epidermoid: 10 (8.7) Intraosseous cavernoma: 19 (16.5) Aneurysmal bone cyst: 4 (3.5) Fibrous dysplasia: 6 (5.2)	Metastasis: 27 (45.8) Malignant hematopoietic tumors (plasmacytoma and lymphoma): 10 (16.9) Basalioma: 3 (5.1) SCC: 15 (25.4) Chordoma: 4 (6.8)	
**Localization, n (%)**			
Frontal	70 (60.9)	25 (42.4)	**0.02**
Temporal	11 (9.6)	10 (16.9)	0.2
Parietal	17 (14.8)	13 (22.0)	0.3
Occipital	8 (7.0)	5 (8.5)	0.8
Suboccipital	9 (7.8)	2 (3.4)	0.3
Skull base	0	4 (6.8)	**0.01**
**Side, n (%)**			
Right	52 (45.2)	21 (35.6)	0.3
Left	44 (38.3)	28 (47.4)	0.3
Midline	17 (14.8)	9 (15.3)	0.99
Bothsided	2 (1.7)	1 (1.7)	0.99
**Initial surgery, n (%)**			
Biopsy	0 (0)	5 (8.5)	**0.004**
Craniotomy	102 (88.7)	52 (88.1)	0.99
Abrasion	13 (11.3)	2 (3.4)	0.09
**Infiltration of meninges, n (%)**			
Yes	29 (25.2)	24 (40.7)	**0.04**
No	86 (74.8)	35 (59.3)	
**Sinus infiltration, n (%)**			
Yes	8 (7.0)	9 (15.3)	0.1
No	107 (93.0)	50 (84.7)	
**Duroplasty, n (%)**			
Yes	30 (26.1)	21 (35.6)	0.2
No	85 (73.9)	38 (64.4)	
**Revisions, n (%)**			
Wound healing disorder	1 (0.9)	3 (5.1)	0.1
Bleeding	2 (1.7)	7 (11.9)	**0.008**

ASA classification: American Society of Anesthesiologists classification; BMI: body mass index; GOS: Glasgow Outcome Scale; KPS: Karnofsky Performance Status Scale; mFI-5: modified 5-Item Frailty Index; mRS: modified Rankin Scale; SD: standard deviation.

The most common presenting symptom was local swelling (n = 104, 59.8%), followed by headache (n = 36, 20.7%) and tenderness (n = 18, 10.3%). Neurological deficits were less frequent, including gait disturbance (n = 9, 5.2%), paresis (n = 7, 4.0%), vertigo (n = 7, 4.0%), and epileptic seizures (n = 4, 2.3%). Five patients (2.9%) were asymptomatic at presentation, all of whom were diagnosed incidentally on imaging performed for unrelated indications. Malignant lesions were significantly associated with reduced general condition (n = 9/59, 15.3% vs. n = 2/115, 1.7%; p = 0.001), headache (n = 6/59, 10.2% vs. n = 30/115, 26.1%; p = 0.02), and dysphagia (n = 3/59, 5.1% vs. n = 0/115; p = 0.04) (Table 2). With respect to neurological symptoms, no significant differences in symptom incidence were observed between lesions confined to the calvarial bone and those demonstrating infiltration of the meninges or brain parenchyma, with the exception of aphasia, which was significantly more frequent in patients with lesions exhibiting meningeal or intracranial involvement (p = 0.03).

**Table 2:** Distribution of presenting symptoms in patients with calvarial lesions, stratified by benign versus malignant histopathological classification.

*Symptoms*	Benign lesion n (%)	Malignant lesion n (%)	p-value
None	4/115 (3.5)	1/59 (1.7)	0.7
Headache	30/115 (26.1)	6/59 (10.2)	**0.02**
Reduced general condition	2/115 (1.7)	9/59 (15.3)	**0.001**
Dysphagia	0/115 (0)	3/59 (5.1)	**0.04**
Paresis	4/115 (3.5)	3/59 (5.1)	0.7
Sensibility disorder	1/115 (0.9)	1/59 (1.7)	0.99
Epileptic seizures	4/115 (3.5)	0/59 (0)	0.3
Aphasia	1/115 (0.9)	2/59 (3.4)	0.3
Local swelling	71/115 (61.7)	33/59 (55.9)	0.5
Tenderness	13/115 (11.3)	5/59 (8.5)	0.8
Gait disorder	3/115 (2.6)	6/59 (10.2)	0.06
Vertigo	6/115 (5.2)	1/59 (1.7)	0.4
Visual disorder	2/115 (1.7)	3/59 (5.1)	0.3

### 3.2 Lesion localization and laterality

Lesions were most frequently located in the frontal bone (n = 95, 54.6%), followed by the parietal (n = 32, 18.4%), temporal (n = 21, 12.1%), and occipital bones (n = 15, 8.6%). Suboccipital involvement was observed in 11 patients (6.3%), while 4 lesions (2.3%) involved the skull base. Malignant lesions showed a significantly higher incidence of skull base involvement (n = 4/59, 6.8%) compared to benign lesions (n = 0/115, *p* = 0.01). Regarding laterality, 73 lesions (42.0%) were right-sided, 72 (41.4%) left-sided, 26 (14.9%) midline, and 3 (1.7%) bilateral, without significant differences between benign and malignant groups.

#### 3.2.1 Radiological characteristics

**Fig. 2:**
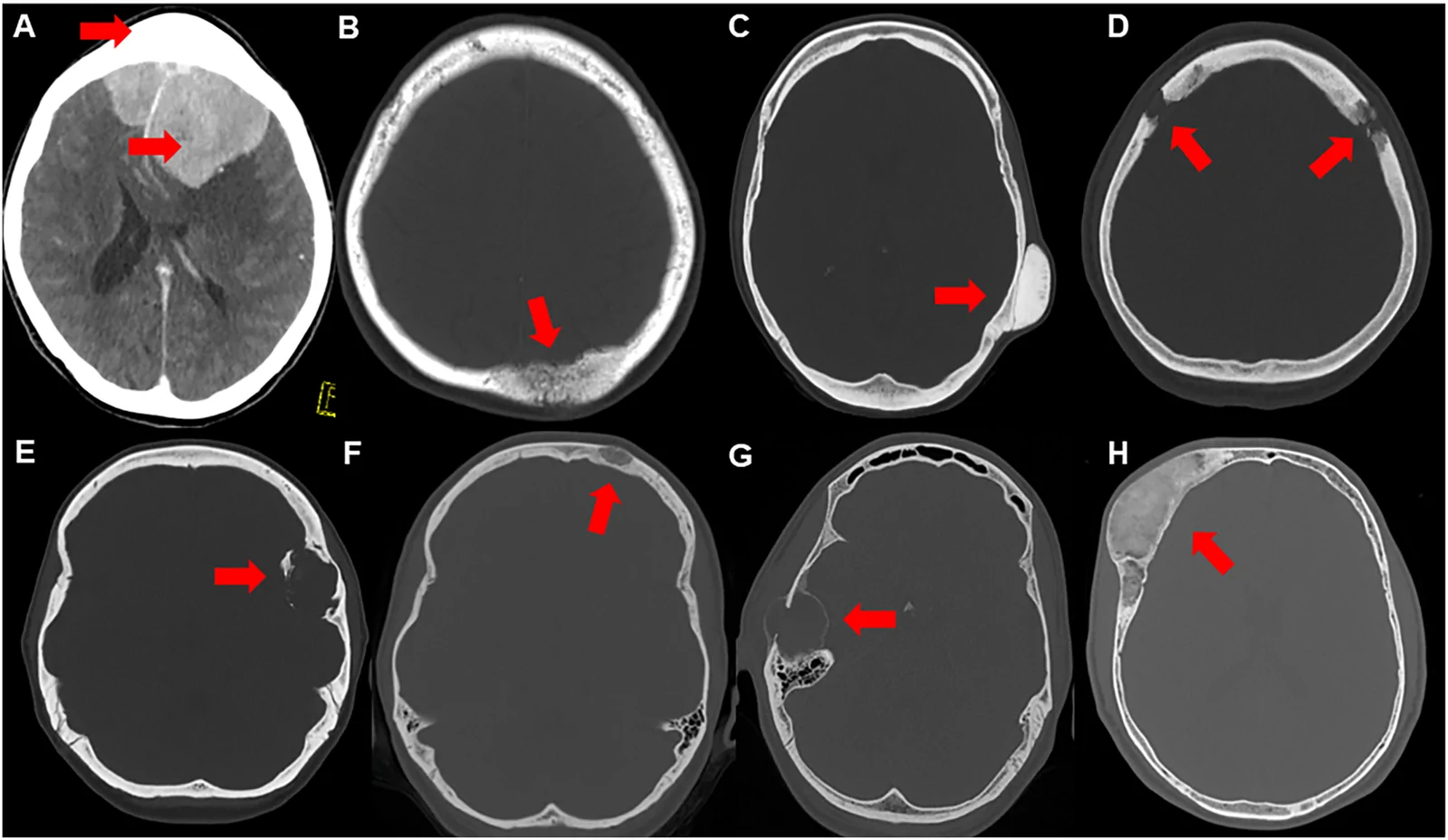
Representative CT features of benign calvarial lesions. (A, B) Intraosseous meningioma demonstrating characteristic bone involvement, (C) osteoma with well-defined osseous architecture, (D) Langerhans cell histiocytosis showing osteolytic bone changes, (E) epidermoid, (F) intraosseous cavernoma, (G) aneurysmal bone cyst, (H) fibrous dysplasia. These examples illustrate the morphological heterogeneity of benign calvarial pathologies encountered in the cohort.

**Fig. 3:**
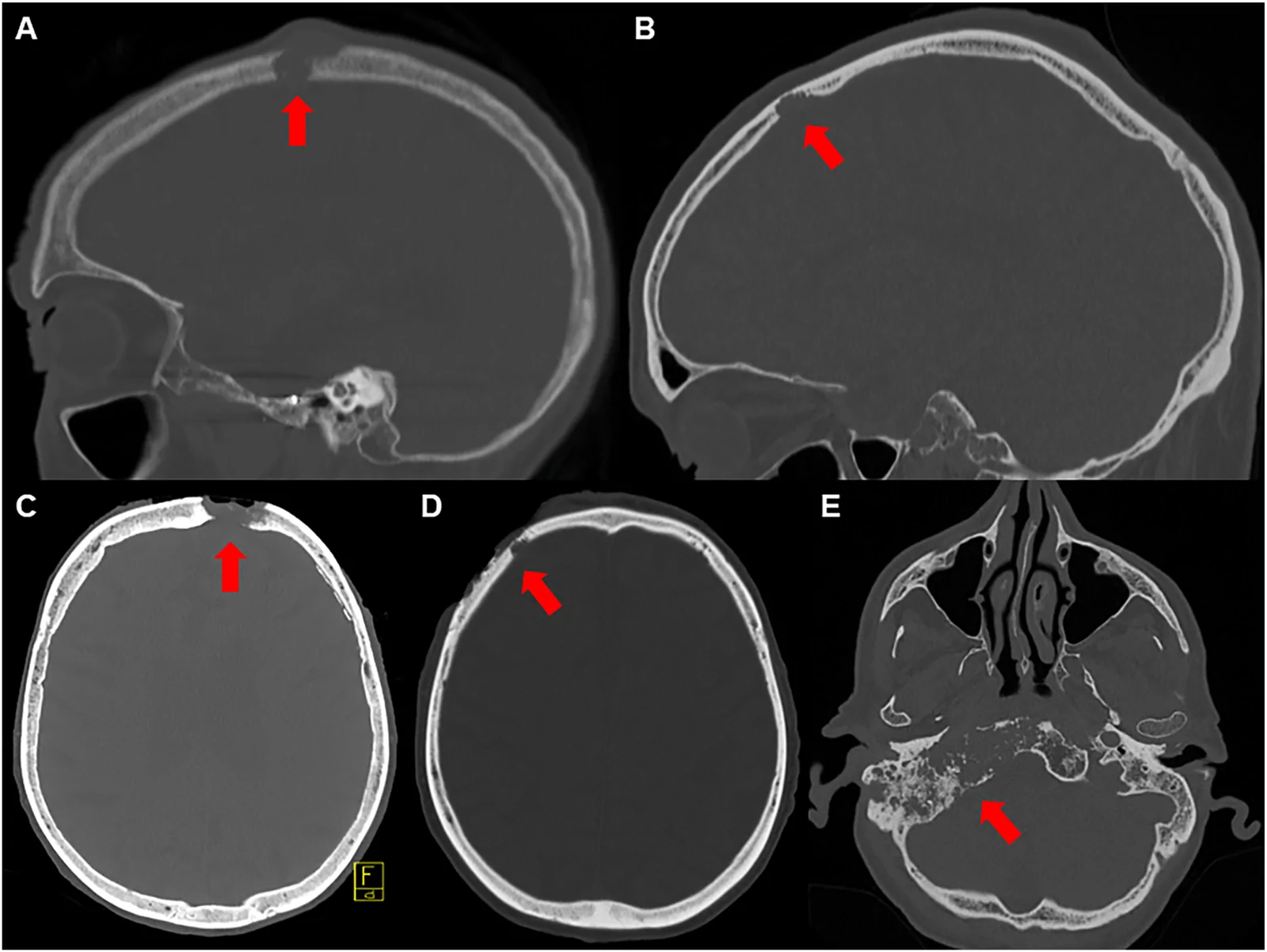
Representative CT features of malignant and infiltrative calvarial lesions. (A) Metastatic lesion (adenocarcinoma of the parotid gland), (B) hematopoietic lesion (plasmacytoma), (C) basal cell carcinoma, (D) squamous cell carcinoma (SCC), (E) chordoma. The images highlight typical patterns of osseous destruction observed in malignant calvarial disease.

On CT imaging, osteolytic morphology was the predominant pattern (n = 116, 66.7%), followed by no pathological osseous changes (n = 28, 16.1%), and osteoblastic lesions (n = 18, 10.3%). Osteolytic morphology was significantly associated with malignant lesions (n = 50/59, 84.7% vs. n = 66/115, 57.4%; p = 0.0003) (Fig. 2, Fig. 3).

MRI analyses were based on all available preoperative and postoperative MRI examinations retrieved from the institutional imaging archive. If a specific imaging examination or sequence was unavailable, the corresponding variable was recorded as missing and excluded only from the respective analysis; no imputation of missing values was performed. The proportion of unavailable imaging sequences was consistently low, with an overall missingness of approximately 15% across all assessed MRI parameters.

MRI demonstrated heterogeneous signal characteristics. On T1-weighted imaging, lesions were hypointense in 43 cases (24.7%), isointense in 56 cases (32.2%), and hyperintense in 52 cases (29.9%). On T2-weighted imaging, hyperintensity predominated (n = 86, 49.4%), followed by isointensity (n = 33, 19.0%) and hypointensity (n = 32, 18.4%). Contrast enhancement was present in 122 evaluable cases (70.1%). No significant differences in MRI signal characteristics or contrast enhancement were observed between benign and malignant lesions (Table 3). Dural infiltration was observed in 53 patients (30.5%) and was significantly more common in malignant lesions (n = 24/59, 40.7% vs. n = 29/115, 25.2%; p = 0.04). Venous sinus infiltration occurred in 17 cases (9.8%), without significant group differences.

**Table 3:** Radiological features of patients with calvarial lesions, stratified by benign versus malignant histopathological classification.

*Parameters*	Benign lesion (n = 115)	Malignant lesion (n = 59)	*p-value*
**CT, n (%)**			
Osteolytic	66 (57.4)	50 (84.7)	**0.0003**
Extension of bony involvement			
*Complete*	10 (15.2)	7 (14.0)	
*Partial*	56 (84.8)	43 (86.0)	0.99

Lesion Number			
*Solitary*	63 (95.5)	31 (62.0)	
*Multiple*	3 (4.5)	19 (38.0)	**<0.0001**

Osteoblastic	15 (13.0)	3 (5.1)	0.1
Mixed osteolytic/osteoblastic	0 (0)	0 (0)	0.99
No osseous changes	25 (21.7)	3 (5.1)	**0.004**
N/A	9 (7.2)	3 (5.1)	0.8

**MRI, n (%)**			

**T1**			
Hypointense	31 (27.0)	12 (20.3)	0.4
Hyperintense	34 (30.0)	18 (30.5)	0.99
Isointense	36 (31.3)	20 (33.9)	0.7
N/A	14 (12.2)	9 (15.3)	0.6

**T2**			
Hypointense	22 (19.1)	10 (16.9)	0.8
Hyperintense	56 (48.7)	30 (50.8)	0.9
Isointense	23 (20.0)	10 (16.9)	0.7
N/A	14 (12.2)	9 (15.3)	0.6

**Contrast dye**			
Yes	80 (69.6)	42 (71.2)	0.9
No	17 (14.8)	6 (10.2)	0.5
N/A	18 (15.6)	11 (18.6)	0.7

### 3.3 Histopathological spectrum

The most frequent histopathological diagnoses were meningioma (n = 30, 17.2%), metastatic lesions (n = 27, 15.5%), Langerhans cell histiocytosis (n = 24, 13.8%), osteoma (n = 22, 12.6%), and intraosseous cavernoma (n = 19, 10.9%).

**Fig. 4:**
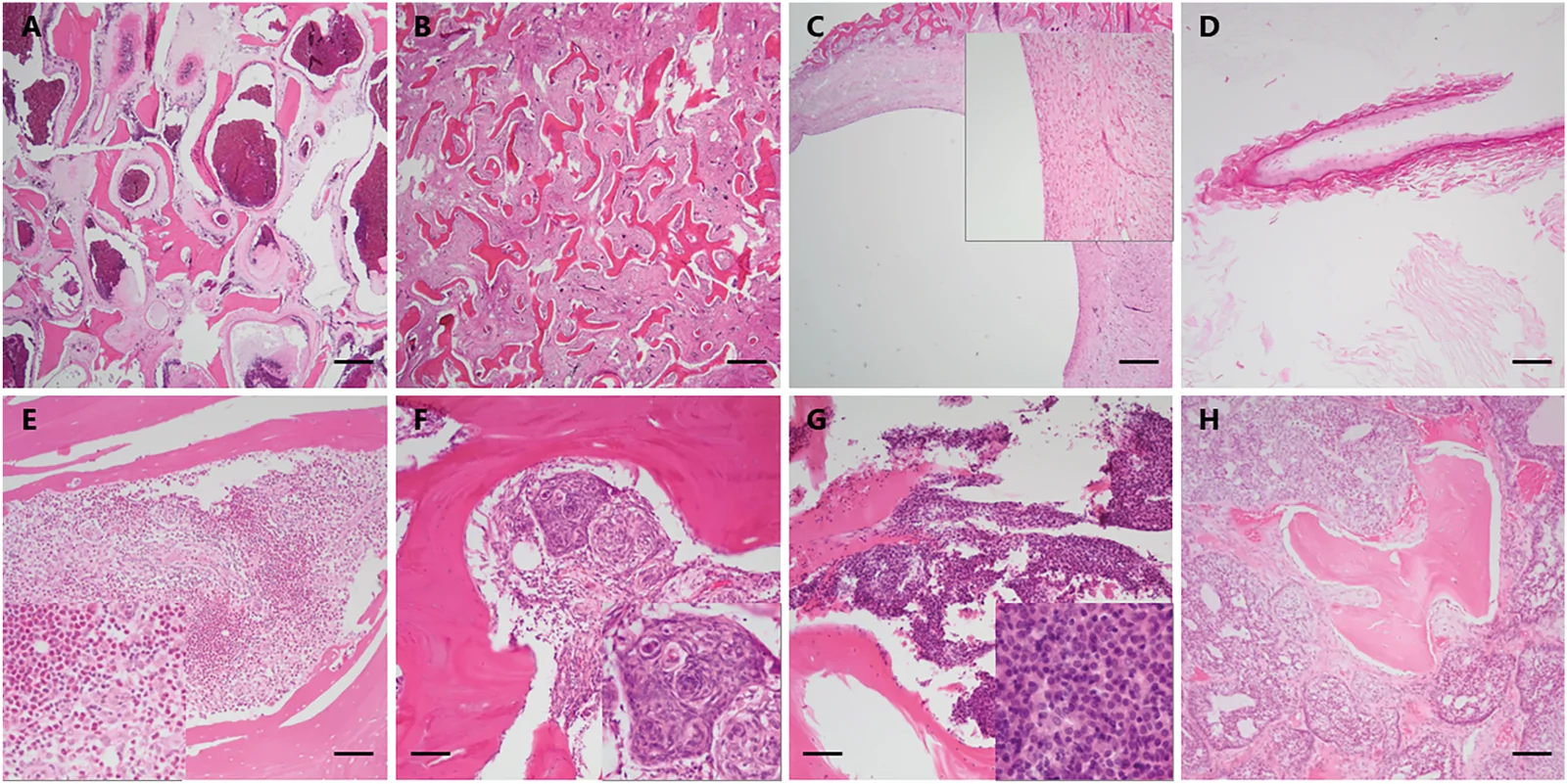
(A) Intraosseous hemangioma showing numerous irregularly shaped, convoluted blood vessels located between bone trabeculae. (B) Fibrous dysplasia characterized by irregularly branching trabeculae of woven bone within a fibrous spindle-cell stroma. (C) Simple bone cyst demonstrating a fibrous cyst wall lacking an epithelial lining. (D) Epidermoid cyst composed of laminated keratin debris shed by an inconspicuous stratified squamous epithelium. (E) Langerhans cell histiocytosis with infiltrates of Langerhans cells accompanied by an inflammatory infiltrate predominantly composed of eosinophils. (F) Infiltrates of a meningioma showing prominent whorl formation. (G) Plasmacytoma with diffuse intraosseous infiltration by sheets of neoplastic plasma cells. (H) Osteolytic metastasis from breast carcinoma. Scale bars: 500 μm (A–C, G); 100 μm (D–F, H).

Among malignant lesions (n = 59), metastatic disease represented the largest subgroup (n = 27, 45.8%), followed by squamous cell carcinoma (n = 15, 25.4%) and hematopoietic malignancies, including plasmacytoma and lymphoma (n = 10, 16.9%). Benign lesions (n = 115) were predominantly meningiomas (n = 30, 26.1%), osteomas (n = 22, 19.1%), Langerhans cell histiocytosis (n = 24, 20.9%), and intraosseous cavernomas (n = 19, 16.5%) (Fig. 4). The histopathological findings of all entities were consistent with their classical morphological features.

For practical clinical reference, we additionally provide a structured table outlining the most important histopathological differential diagnoses and their defining characteristics (Table 4).

**Table 4:** Major histopathological differential diagnoses of calvarial lesions and their defining microscopic features, stratified into benign and malignant entities.

Calvarial Lesions	Common histopathological features
***Benign Lesions***	
***Neoplastic/True Bone Tumors***	
**Osteoma**	Proliferation of bland lamellar or woven bone with cortical or trabecular architecture
**Intraosseous hemangioma**	Intraosseous proliferation of irregularly shaped convoluted capillary or cavernous blood vessels
**Fibrous dysplasia**	Fibrous stroma with spindle cells and irregular, branching trabeculae of woven bone (“Chinese letters”)
**Aneurysmal bone cyst (ABC)**	Septate blood-filled cystic lesion containing fibroblasts, multinucleated giant cells, and woven bone; primary ABC associated with chromosomal translocation t(16; 17)(q22; p13)
**Simple bone cyst**	Pseudocyst with a thin fibrous cyst wall lacking an epithelial lining
***Cystic/Epithelial Lesions***	
**Epidermoid cyst**	Cyst lined by inconspicuous stratified squamous epithelium shedding masses of layered keratin without any evidence of skin appendages
**Dermoid cyst**	Cyst lined by inconspicuous stratified squamous epithelium shedding masses of layered keratin, skin appendages may be present
***Histiocytic/Hematopoietic Lesions***	
**Langerhans cell histiocytosis**	Large round to oval Langerhans cells with reniform nuclei, accompanied by an inflammatory infiltrate dominated by eosinophils and occasional multinucleated giant cells
**Systemic/Metabolic/Granulomatous**	
**Paget's disease of bone**	Disorganized lamellar and woven bone with irregular cement lines, prominent osteoblastic rimming of thickened trabeculae and areas of bone resorption with large multinucleated osteoclasts
**Sarcoidosis**	Non-caseating epithelioid granulomas with multinucleated Langhans-type giant cells and peripheral lymphocytic rim
**Tuberculosis**	Caseating epithelioid granulomas with central necrosis, multinucleated Langhans-type giant cells and peripheral lymphocytic rim
***Other Benign Tumors***	
**Meningioma**	Intraosseous meningothelial infiltrates exhibiting whorled, nodular or fascicular growth patterns

***Malignant lesions***	
***Primary Bone/Hematopoietic Malignancies***	
**Chordoma**	Lobular growth pattern with chord-like arrangement of epitheloid tumor cells of sometimes physaliphorous appearance embedded in a mucoid matrix
**Plasmacytoma**	Sheets of plasma cells at varying stages of maturation, sometimes showing atypia, with scant intervening stroma
**Primary bone lymphoma**	Most commonly diffuse large B-cell lymphoma (DLBCL): Diffuse infiltrates of atypical B-lymphoid cells with enlarged nuclei and prominent nucleoli
***Secondary malignant involvement***	
**Metastasis**	Infiltrative intraosseous proliferation of mostly atypical epithelial cells, forming nests or sheets, sometimes with associated osteolysis and/or reactive bone formation, depending on the primary tumor
**Direct extension (*per continuitatem*) of adjacent malignancies**	Direct invasion of bone by an adjacent malignant neoplasm, most commonly cutaneous squamous cell carcinoma or rarely basal cell carcinoma, with histological features reflecting the primary tumor

### 3.4 Surgical management and complications

Indications for surgical intervention were based on interdisciplinary clinical decision-making and included one or more of the following: (1) radiological suspicion of malignancy requiring histopathological confirmation, (2) symptomatic lesions (e.g., pain, swelling, neurological deficits), (3) progressive growth on serial imaging, or (4) lesions requiring resection for local control or reconstruction.

Initial surgical management consisted primarily of craniotomy with lesion excision (n = 154, 88.5%), followed by lesion abrasion without craniotomy (n = 15, 8.6%) and biopsy only (n = 5, 2.9%) (Fig. 1). Biopsy-only procedures were significantly more frequent in malignant lesions (n = 5/59, 8.5% vs. n = 0/115, *p* = 0.004). Gross total resection (GTR), defined as the absence of residual lesion on postoperative imaging (postoperative lesion volume = 0 cm^3^), was achieved in 144 of 174 patients (82.8%), including 96 of 115 benign lesions (83.5%) and 48 of 59 malignant lesions (81.3%), without a significant difference between groups (p = 0.8).

Reconstructive strategies were individualized according to defect characteristics and intraoperative findings. Dural reconstruction was performed either by primary dural closure or duroplasty, with duroplasty required in 51 patients (29.3%). Cranial reconstruction was predominantly performed using PMMA cranioplasty, whereas superficial calvarial defects or limited bone involvement did not require reconstruction. Due to the individualized nature of reconstructive decision-making, no systematic differences according to histopathological entity or defect size could be established.

In total, 201 neurosurgical procedures, including revision surgeries, were performed. Postoperative complications after the initial operation occurred in 13 patients (7.5%), comprising wound infections (n = 8, 4.6%) and postoperative hemorrhage (n = 5, 2.9%). Overall, 20 second procedures and 7 third procedures were performed. Of the second procedures, 13 (65.0%) represented complication-related revision surgeries following the initial operation, whereas the remaining procedures comprised one laminectomy performed for distant tumor progression, two stereotactic procedures, and four craniotomies performed for tumor recurrence. Among the seven third procedures, three (42.9%) were revision surgeries following the second procedure, while the remaining four craniotomies were performed for recurrent disease, including two patients with a first recurrence and two patients with a second recurrence. The median time to complication-related revision surgery was 56.0 days, whereas recurrence-related reoperations occurred substantially later, with a median time to recurrence of 1660 days for benign lesions and 973.5 days for malignant lesions. Revision surgery was significantly more frequent in malignant lesions, particularly due to postoperative bleeding (n = 7/59, 11.9% vs. n = 2/115, 1.7%; p = 0.008).

### 3.5 Functional outcome and follow-up

Functional outcomes improved significantly following surgery. Mean KPS increased from 87.4 ± 9.7 preoperatively to 94.5 ± 10.6 postoperatively (*p* < 0.0001), and mRS improved from 1.1 ± 0.7 to 0.4 ± 0.8 (*p* < 0.0001). Notably, the observed functional improvement in patients with benign lesions primarily reflects resolution of local symptoms (e.g., pain, mass effect, cosmetic impairment) rather than recovery from severe neurological deficits, as most patients were functionally preserved preoperatively. Surgical indications in these cases were therefore frequently driven by symptomatic burden or progressive lesion growth rather than marked neurological decline. No significant change was observed in GOS scores (*p* = 0.6). Improvements were more pronounced in patients with benign lesions. The median follow-up duration was 461 days (15.1 months) overall, comprising a median follow-up of 315 days (10.3 months) in patients with benign lesions and 504 days (16.6 months) in those with malignant lesions. During follow-up, no deaths occurred among patients with benign lesions, whereas 12 of 59 patients (20.3%) with malignant lesions died, reflecting the substantially poorer prognosis of malignant calvarial disease. Overall, recurrence occurred in 6 of 174 patients (3.4%), comprising 2 of 115 patients (1.7%) with benign lesions and 4 of 59 patients (6.8%) with malignant lesions. The median time to recurrence was 1660 days for benign lesions and 973.5 days for malignant lesions. Although recurrence was numerically more frequent in malignant lesions, this difference did not reach statistical significance (p = 0.2).

### 3.6 Predictors of malignancy

Variables identified as significant in the univariate analyses were entered into separate multivariate regression models for preoperative predictors (age, dural infiltration, osteolytic lesion on CT, multiple osteolytic lesions, mFI-5, ASA score, preoperative KPS, preoperative GOS, and preoperative mRS) and postoperative outcomes (postoperative complications, postoperative KPS, postoperative GOS, and postoperative mRS), as summarized in Table 5.

**Table 5:** Univariate and multivariate regression analyses for predictors of malignancy.

Variable	Univariate OR	95% CI	p-value	Multivariate OR	95% CI	p-value
***Preoperative***						
Multiple osteolytic lesions	6.21	3.05–12.65	**<0.0001**	5.34	2.35–12.14	**<0.0001**
ASA score	2.01	1.48–2.74	**<0.0001**	1.68	1.27–2.22	**0.0002**
Age (per year)	1.04	1.02–1.06	**<0.0001**	1.03	1.01–1.05	**0.0004**
mRS (preoperative)	2.18	1.41–3.37	**0.0006**	1.72	1.14–2.60	**0.01**
KPS (preoperative)	0.67	0.57–0.79	**<0.0001**	0.82	0.69–0.98	**0.02**
Osteolytic lesion on CT	3.87	1.87–8.02	**0.0003**	1.94	0.87–4.34	0.10
mFI-5	1.72	1.35–2.20	**<0.0001**	1.18	0.85–1.64	0.31
GOS (preoperative)	0.43	0.27–0.69	**<0.0001**	0.76	0.47–1.23	0.27
Dural infiltration	2.05	1.03–4.09	**0.04**	1.47	0.68–3.18	0.33
Sex (male)	1.08	0.63–1.86	0.78	—	—	—
Side	1.06	0.82–1.37	0.66	—	—	—
Sinus infiltration	1.83	0.78–4.31	0.16	—	—	—
Contrast enhancement on MRI	1.42	0.73–2.77	0.30	—	—	—
BMI	0.97	0.92–1.03	0.35	—	—	—
Extent of osteolysis	1.29	0.86–1.94	0.22	—	—	—

***Postoperative***						
mRS (postoperative)	2.74	1.78–4.22	**<0.0001**	1.78	1.02–3.11	**0.04**
KPS (postoperative)	0.61	0.51–0.73	**<0.0001**	0.88	0.71–1.09	0.24
GOS (postoperative)	0.39	0.24–0.63	**<0.0001**	0.81	0.47–1.40	0.45
Complications	2.86	1.62–5.05	**0.0003**	1.51	0.73–3.11	0.27

ASA: American Society of Anesthesiologists Physical Status Classification; BMI: body mass index; cCT: cranial computed tomography; CI: confidence interval; GOS: Glasgow Outcome Scale; KPS: Karnofsky Performance Status; mFI-5: modified 5-Item Frailty Index; MRI: magnetic resonance imaging; mRS: modified Rankin Scale; OR: odds ratio.

Univariate analysis identified several preoperative variables significantly associated with malignant lesions, including increasing age (p < 0.0001), dural infiltration (p = 0.04), osteolytic morphology on CT (p = 0.0003), the presence of multiple osteolytic lesions (p < 0.0001), higher modified 5-Item Frailty Index (mFI-5) scores (p < 0.0001), higher ASA classifications (p < 0.0001), lower preoperative Karnofsky Performance Status (KPS) scores (p < 0.0001), lower preoperative Glasgow Outcome Scale (GOS) scores (p < 0.0001), and higher preoperative modified Rankin Scale (mRS) scores (p = 0.0006). In the multivariate analysis restricted to preoperative variables, the presence of multiple osteolytic lesions on CT remained the strongest independent predictor of malignancy (p < 0.0001). In addition, increasing age (p = 0.0004), higher ASA classification (p = 0.0002), lower preoperative KPS (p = 0.02), and higher preoperative mRS (p = 0.01) remained independently associated with malignant lesions, whereas dural infiltration, osteolytic morphology alone, mFI-5, and preoperative GOS did not retain statistical significance.

Postoperative outcome analyses demonstrated that postoperative complications (p = 0.0003), lower postoperative KPS (p < 0.0001), lower postoperative GOS (p < 0.0001), and higher postoperative mRS (p < 0.0001) were significantly associated with malignant lesions in univariate analyses. In the separate postoperative multivariate analysis, only postoperative mRS remained independently associated with malignancy (p = 0.04), whereas postoperative complications, postoperative KPS, and postoperative GOS did not retain statistical significance.

## 4 Discussion

Calvarial lesions represent an uncommon but clinically relevant spectrum of pathologies that challenge neurosurgeons due to their heterogeneous biological behavior and variable radiological appearance. These lesions are highly diverse, ranging from metastases (Gaetani et al., 2005; Zhou et al., 2025), meningiomas (Al-Khawaja et al., 2007; Elder et al., 2007; Sasaki et al., 2021), Langerhans cell histiocytosis (Allen et al., 2018), plasmacytomas (Harapan et al., 2025), cavernomas (Alexiou et al., 2022; AlMakadma and Alkhani, 2013), and hemangiomas (Krishnan et al., 2024) to numerous other neoplastic and non-neoplastic entities. In this large single-center cohort, we present a comprehensive multidisciplinary analysis integrating clinical presentation, radiological characteristics, histopathological diagnosis, and neurosurgical management over more than a decade. To our knowledge, this study constitutes one of the most extensive neurosurgical series to date that systematically combines these complementary domains within a unified analytical framework, thereby addressing a relevant gap in the existing literature.

A central concept of the present work is the pragmatic comparison between benign and malignant calvarial lesions across all stages of evaluation and treatment. While acknowledging that the biological behavior of calvarial lesions often exists along a continuum rather than within rigid categories, this classification provides a clinically meaningful structure for preoperative risk stratification and surgical planning. Our findings underscore that patients harboring malignant lesions were significantly older and exhibited higher frailty and ASA scores, reflecting a greater systemic disease burden. These observations align with prior reports suggesting that calvarial involvement by malignant disease frequently occurs in the context of advanced systemic pathology rather than isolated cranial disease (Shuman et al., 2025).

A clinically relevant observation relates to the relationship between lesion extent and neurological presentation. Despite the intuitive assumption that meningeal or intracranial involvement would invariably translate into a higher incidence of neurological deficits, our analysis demonstrated comparable rates of most neurological symptoms between lesions confined to the calvarial bone and those exhibiting infiltration of adjacent structures. The only exception was aphasia, which was significantly more frequent in patients with meningeal or brain involvement. This finding suggests that neurological symptomatology in calvarial lesions may be influenced not solely by anatomical extension but also by factors such as lesion location and growth dynamics. Clinically, this underscores the importance of comprehensive imaging assessment even in patients with minimal or nonspecific neurological findings. An interesting and seemingly counterintuitive finding was the higher frequency of headache in patients with benign lesions. This may reflect differences in growth dynamics rather than biological aggressiveness. Slowly growing benign lesions may cause progressive stretching of the richly innervated periosteum, resulting in persistent localized headache, whereas malignant lesions often present with infiltrative bone destruction, systemic manifestations, or neurological deficits that may overshadow headache as the predominant symptom. Accordingly, headache alone should not be considered a reliable indicator of lesion biology, underscoring the importance of comprehensive radiological and clinical assessment.

From a radiological perspective, CT-based bone morphology provided important diagnostic clues. While osteolytic changes were significantly associated with malignant lesions in univariate analysis, the presence of multiple osteolytic lesions emerged as the strongest independent predictor of malignancy in multivariate analysis. This finding highlights the continued relevance of CT imaging in the initial assessment of calvarial lesions, particularly for evaluating bone destruction patterns (Gökçe and Beyhan, 2025; Khodarahmi et al., 2021; Garfinkle et al., 2011), while underlining the diagnostic value not merely of osteolysis itself but of its distribution pattern within the calvarium. A particular strength of the present study is the more detailed characterization of osteolytic lesions, which were systematically subclassified according to lesion number (solitary vs. multiple) and the extent of bony involvement. Osteolytic lesions were differentiated into partial destruction, limited to one or more components of the calvarial structure (internal table, diploë, or external table), and complete destruction, defined as full-thickness involvement of all calvarial layers. More detailed radiological grading systems, such as the Lodwick classification (Benndorf et al., 2022), which evaluate patterns of bone destruction based on descriptors including the type of osteolysis (geographic with margin characteristics, moth-eaten, or permeative), cortical penetration, the presence of a sclerotic rim, and the presence of an expanded shell, were not systematically applied in the present study. Similarly, the Modified Lodwick–Madewell Grading System, which refines the assessment of lytic bone lesions by incorporating additional patterns associated with increasing biological activity and malignancy risk, was not applied, as it was primarily developed for osseous lesions in general rather than specifically for calvarial pathology (Caracciolo et al., 2016). In clinical practice, calvarial lesions frequently display mixed morphological features, which may limit the consistent applicability of these detailed descriptors in routine neurosurgical decision-making. Moreover, a recent study has demonstrated poor interobserver reliability and only moderate intra-observer reproducibility for commonly used radiographic classifications of radiolucent bone lesions (Willenbring et al., 2025). Our simplified approach focusing on lesion number and the extent of calvarial destruction therefore offers a pragmatic and reproducible framework for neurosurgical practice.

In contrast, MRI signal characteristics on T1-and T2-weighted sequences and contrast enhancement did not significantly differ between benign and malignant entities, emphasizing the limited specificity of MRI signal intensity alone for biological classification. CT and MRI should therefore be regarded as complementary modalities capable of demonstrating distinct yet clinically pertinent features, collectively enabling refinement of a focused differential diagnosis and guiding appropriate management (Mitra et al., 2016). These results reinforce the concept that imaging findings should be interpreted in an integrated manner, rather than in isolation, and that radiological suspicion must ultimately be corroborated by histopathological examination.

Histopathological analysis defined the broad spectrum of calvarial pathology encountered in neurosurgical practice. Benign lesions were dominated by meningiomas, osteomas, Langerhans cell histiocytosis, and intraosseous cavernomas, whereas metastatic disease constituted the largest subgroup among malignant lesions. The spectrum of histopathological entities identified in our cohort broadly reflects those reported in prior smaller series (Nasi-Kordhishti et al., 2021; Ozgiray et al., 2016; Karatas et al., 2023), while the present study extends existing knowledge by correlating histopathological diagnosis with detailed radiological features, surgical strategy, and outcome metrics. Importantly, histopathology served as the definitive reference standard for lesion classification, underscoring its indispensable role in guiding postoperative management and adjuvant therapy.

Surgical management strategies reflected both lesion biology and patient-related factors. Preoperative imaging played an important role not only in lesion characterization but also in surgical planning. Gross total resection was pursued whenever anatomically feasible, while the decision for biopsy or limited surgical intervention was individualized based on radiological suspicion of malignancy, lesion extent, anatomical constraints, and the anticipated morbidity of complete resection. Thus, imaging findings contributed to determining the most appropriate balance between diagnostic certainty, oncological control, and surgical safety. Gross total resection was achievable in the majority of cases (82.8%) and was associated with favorable functional outcomes. Revision surgeries were significantly more frequent in malignant lesions. These findings highlight the necessity of tailoring surgical aggressiveness to both anticipated biological behavior and overall patient condition, rather than pursuing uniform resection strategies. Functional outcomes improved significantly following surgery, especially in patients with benign lesions, as reflected by improvements in KPS and mRS scores. While malignant lesions were associated with higher complication and recurrence rates, surgical intervention nonetheless contributed to symptom control and histological clarification. Recurrence occurred predominantly in malignant and infiltrative lesions, underscoring the importance of close follow-up and interdisciplinary collaboration with oncology and radiation therapy services.

Beyond conventional CT and MRI, several emerging diagnostic techniques may further complement the evaluation of calvarial lesions, although evidence remains limited. PET/CT may be useful in selected patients with suspected metastatic disease or an unknown primary tumor by combining metabolic information with whole-body staging. However, dedicated studies investigating PET/CT specifically in calvarial lesions are scarce. One large retrospective series demonstrated that inclusion of the calvarium in whole-body FDG-PET/CT identified unsuspected calvarial metastases in a small proportion of oncological patients, although these findings rarely altered clinical management (Tasdemir et al., 2014). Likewise, molecular profiling and liquid biopsy are increasingly incorporated into precision oncology but currently have only a limited and largely investigational role in isolated calvarial lesions, with no dedicated studies specifically evaluating their diagnostic or therapeutic utility in this patient population. CT-guided percutaneous biopsy has also emerged as a valuable minimally invasive diagnostic option for indeterminate calvarial lesions, particularly when imaging findings are inconclusive or metastatic disease is suspected. A recent study has demonstrated high diagnostic accuracy with a favorable safety profile, making it a useful adjunct in selected patients prior to definitive treatment (Sundararajan et al., 2022).

Several limitations must be acknowledged when interpreting the results of this study. First, the retrospective design introduces the potential for selection bias, incomplete data capture, and variability in diagnostic and therapeutic approaches over the extended study period. Surgical strategies, imaging protocols, and adjuvant treatment paradigms evolved over time, which may have influenced outcomes. Second, although this represents a large cohort for a rare disease entity, subgroup analyses – particularly for less frequent histopathological diagnoses – remain limited by small sample sizes, restricting the statistical power to detect entity-specific differences. Third, the benign–malignant classification, while clinically pragmatic, may oversimplify the biological complexity of certain lesions that exhibit intermediate or variable behavior. Fourth, functional outcome measures were derived from routine clinical documentation and may not fully capture subtle cognitive, cosmetic, or quality-of-life outcomes that are relevant in calvarial surgery. Fifth, as the present cohort was derived primarily from a neurosurgical department, it reflects lesions requiring neurosurgical evaluation or treatment. Certain calvarial pathologies – such as plasmacytomas or systemic malignancies with skull involvement – may initially be managed by other specialties and treated primarily with systemic therapy, which may lead to underrepresentation of these entities and limits conclusions regarding the overall epidemiology of calvarial lesions. Additionally, the absence of a non-operative comparator group precludes direct assessment of imaging features in lesions managed conservatively and limits conclusions regarding optimal thresholds for surgical versus non-surgical management. Future studies incorporating both operative and non-operative cohorts are warranted to better define management algorithms across the full disease spectrum. Finally, as a single-center study, the generalizability of our findings to other institutions with different surgical expertise may be limited. Despite these limitations, the present study provides a robust, multidisciplinary overview of calvarial lesions in neurosurgical practice and offers clinically relevant insights into the interplay between imaging, histopathology, and surgical management. Future prospective, multicenter studies incorporating standardized imaging protocols and patient-reported outcome measures may further refine risk stratification and optimize individualized treatment strategies for this diverse group of lesions.

## 5 Conclusions

In this large multidisciplinary cohort, calvarial lesions demonstrated a broad clinical and histopathological spectrum. The presence of multiple osteolytic lesions on CT emerged as the strongest independent radiological predictor of malignancy, while neurosurgical treatment resulted in significant functional improvement. These findings emphasize the importance of careful preoperative imaging assessment, as the suspected biological behavior of a lesion may directly influence surgical planning, including the extent of resection, and the indication for biopsy.

Distinguishing between likely benign and malignant lesions prior to surgery is clinically relevant for tailoring operative aggressiveness in the context of the patient's systemic condition and overall prognosis. While benign lesions may be treated with curative resection, lesions suspicious for malignancy – especially in the presence of multiple osteolytic lesions or systemic disease – may require a more individualized strategy integrating diagnostic confirmation and systemic therapy considerations.

A key strength of this study is its multidisciplinary design integrating neurosurgical, radiological, and histopathological perspectives, reflecting real-world clinical decision-making. By applying a pragmatic benign–malignant framework across imaging, surgery, and outcomes, this work provides a clinically applicable perspective that may support more individualized management of calvarial lesions in routine neurosurgical practice.

## Declaration of competing interests

The authors declare that they have no known competing financial interests or personal relationships that could have appeared to influence the work reported in this paper.
